# Supplementary material for: Chemical Components of Dufour’s and Venom Glands in Camponotus japonicus (Hymenoptera, Formicidae)
Source: Insects. 2023 Jul 24;14(7):664. doi: 10.3390/insects14070664 (PMC10380308; doi:10.3390/insects14070664)
Supplement: Supplementary file 1 [file insects-14-00664-s001.zip › insects-2507564-Figures.pdf]

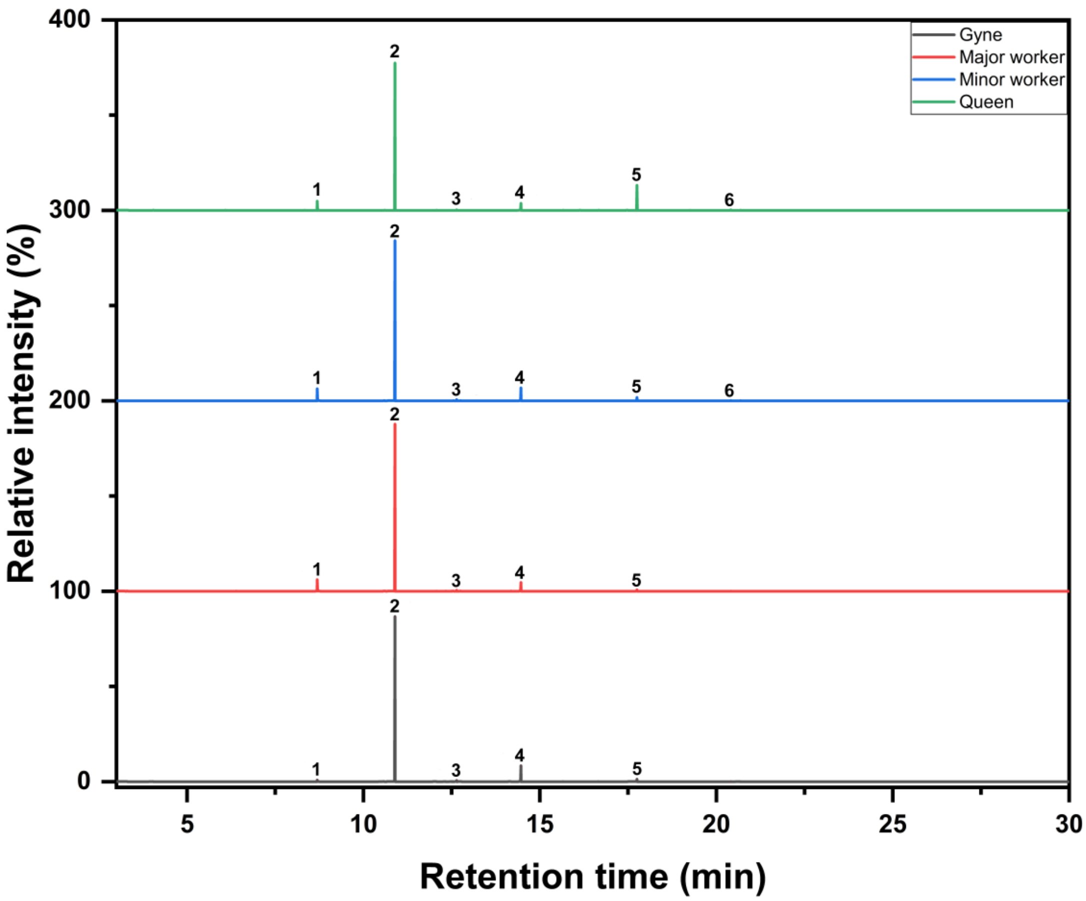

Figure S1: GC–MS total ion current chromatograms of chemical components derived from the Dufour's gland in different castes of *C. japonicus* (SPME method). The peak numbers correspond to compound numbers in Table 1.

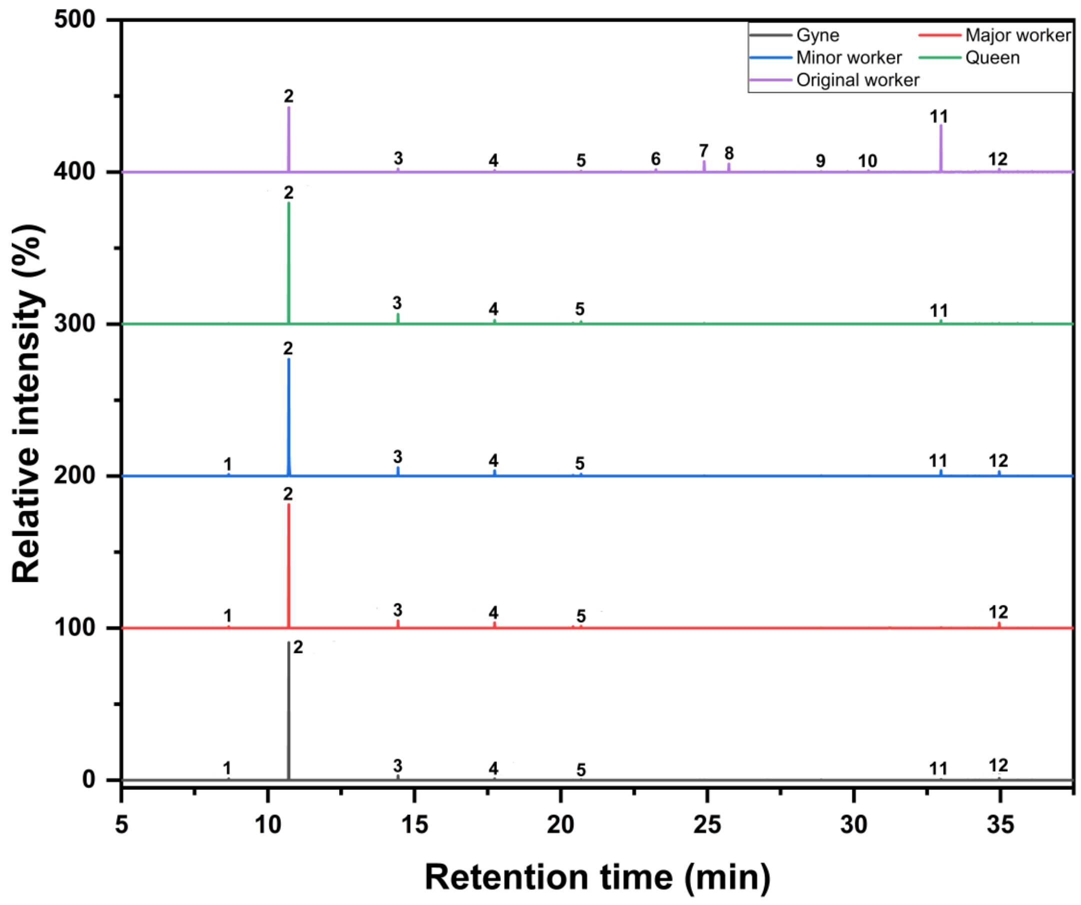

Figure S2: GC–MS total ion current chromatograms of chemical components derived from Dufour's gland in different castes of *C. japonicus* (hexane solution method). The peak numbers correspond to compound numbers in Table 2.

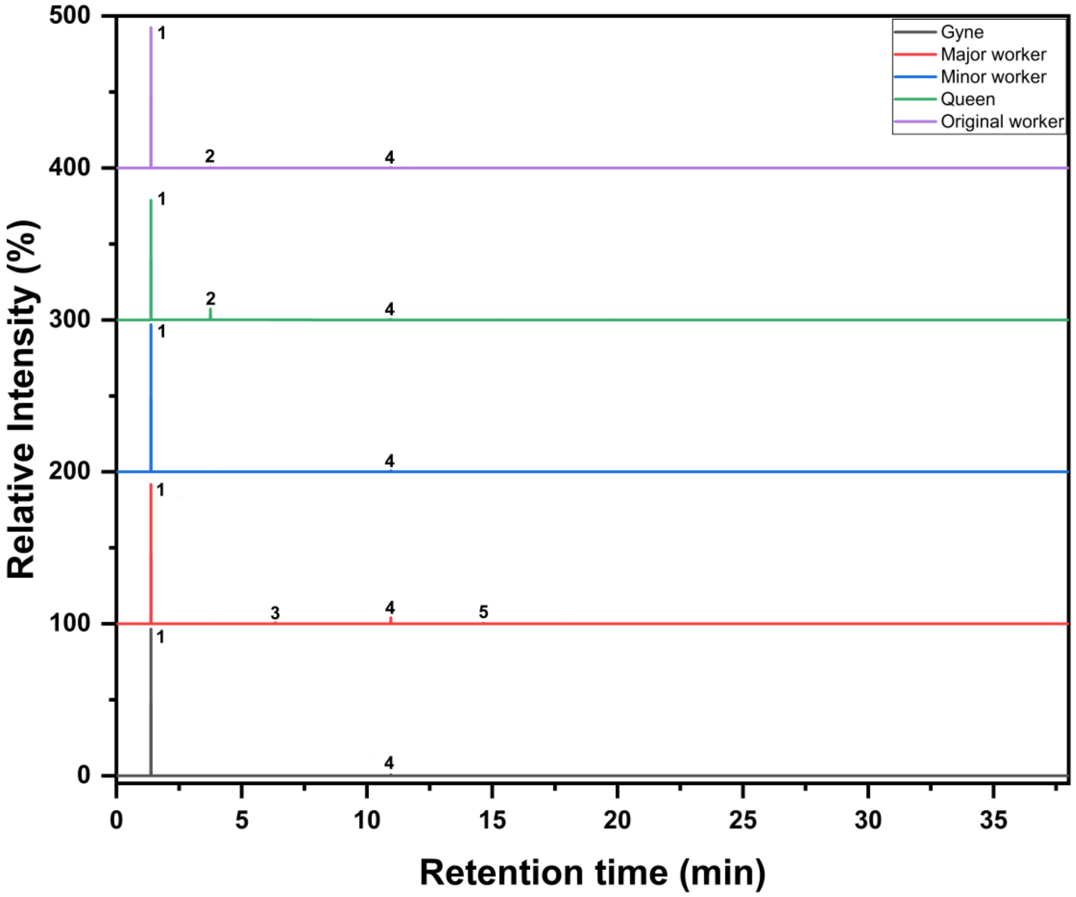

Figure S3: GC–MS total ion current chromatograms of chemical components derived from the venom gland in different castes of *C. japonicus* (SPME method). The peak numbers correspond to compound numbers in Table 3.

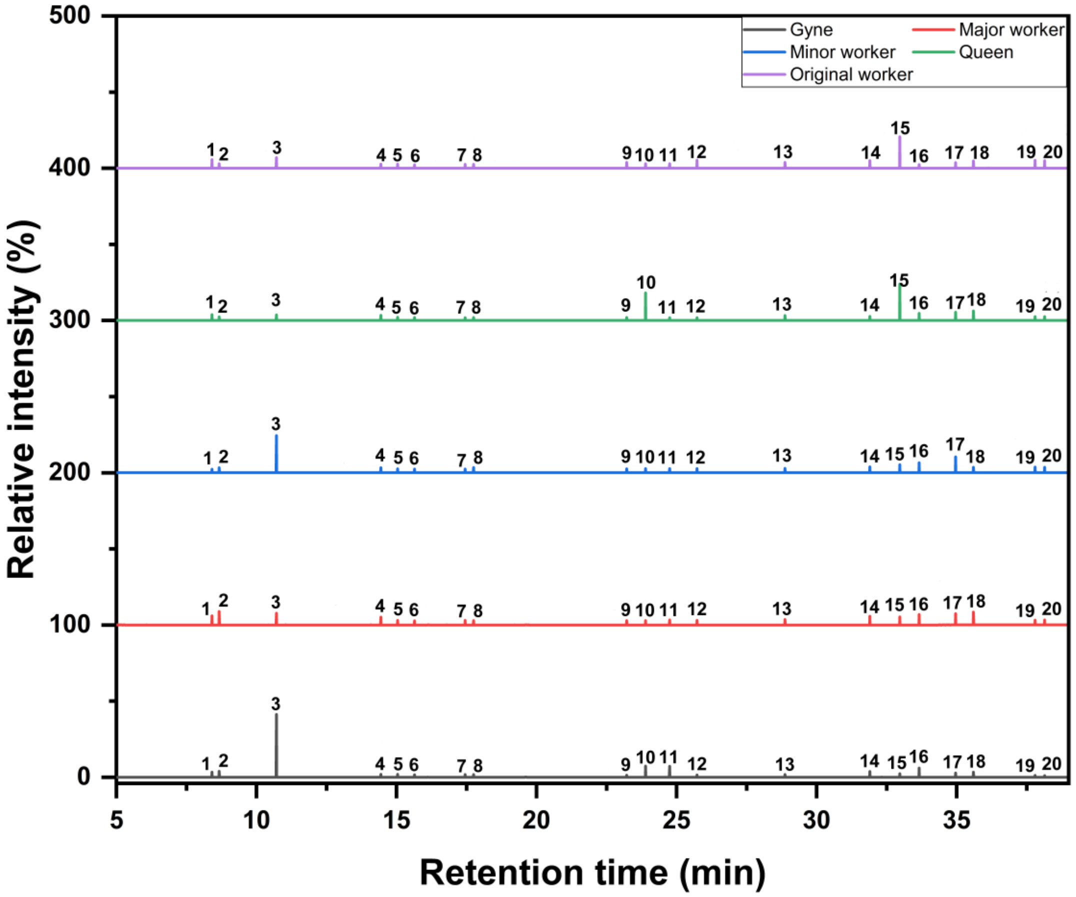

Figure S4: GC–MS total ion current chromatograms of chemical components derived from the venom gland in different castes of *C. japonicus* (hexane solution). The peak numbers correspond to compound numbers in Table 4.
