# Supplementary material for: Chemical Components of Dufour’s and Venom Glands in Camponotus japonicus (Hymenoptera, Formicidae)
Source: Insects. 2023 Jul 24;14(7):664. doi: 10.3390/insects14070664 (PMC10380308; doi:10.3390/insects14070664)
Supplement: Supplementary file 1 [file insects-14-00664-s001.zip › insects-2507564-Table S1.pdf]

Table S1 Summary on the chemical components of Dufour's gland in *Camponotus* ant species

Species and castes	Origin	Main compound (%)	Others	Reference
C. pennsylvanicus, workers	USA	n-Undecane (C_{11})		Ayre and Blum (1971)
C. americanus, workers	USA	n-Undecane (C_{11}), >90%	four alkanes and two alkenes	
C. japonicus, workers	Japan	n-Undecane (C_{11}), 97%	small amount of n-decane, 3-methyl-decane and n-tridecane; trace n-dodecane	Hayashi and Komae (1980)
C. obscuripes, worker	Japan	n-Undecane (C_{11}), 96%		
C. herculeanus, workers	Sweden	n-Tridecane (C_{13})	contains a mixture of 29 similar substances, with five others unidentified, but with only two alkanols and one alkanone and no farnesyl acetate	Bergström and Löfgvist (1972)
C. ligniperda, workers	Sweden	n-Tridecane (C_{13})	contains 37 identified and six unidentified substances, consisting of nalkanes, alkenes, methyl-branched alkanes, nalcohols, 2-alkanones, alkyl and alkenyl acetates, farnesene and farnesyl acetate	
C. intrepidus, workers	Australia	n-Tridecane (C_{13}), 30%	n-undecane (25%), n-decane(0.16%), 5-methylundecane (0.05%), 3-methyl-undecane (5%), n-dodecane (2%), 5-methyl-dodecane (0.05%), 3-methyl-dodecane (0.05%), n-tridecene (0.05%), 5-methyl-tridecane (1.4%), 3-methyl-tridecane (4%), n-tetradecane (1.3%), n-pentadecene (0.05%), n-pentadecane (21%), 5-methyl-pentadecane (0.7%), 3-methyl-pentadecane (0.7%), n-hexadecane (0.2%), 5-methyl-hexadecane (0.05%), 3-methyl-hexadecane (6.5%), n-heptadecane (2.5%), dodec-1-ene (0.05%)	Brophy *et al.* (1973)
C. thoracicus fellah, workers	Israel	n-Undecane (C_{11}), 92%	n-decane (2.5%), n-dodecane (1%), tridecane (1.5%), n-tridecane (4.5%), n-pentadecane (0.5%), n-heptadecane (1%)	Hefetz and Orion (1982)
C. thoracicus sanctoides, workers	Israel	n-Undecane (C_{11}), 75%	Tridecane (11%), pentadecane (4%), Unknown (9.5%); trace n-decane, n-dodecane, n-tetradecane, n-pentadecane, and n-heptadecane	
C. sericeus, workers	Israel	n-Tridecane (C_{13}), 70%	n-undecane (3.4%), n-dodecane (1.8%), 7-methyltridecane (9%), 5-methyltridecane (9%), n-tetradecane (0.8%), n-pentadecane (10%), n-hexadecane (1.5%), heptadecene (3.1%), n-heptadecane (2.8%); trace pentadecene.	
C. lateralis rebeccae, workers	Israel	n-Tridecane (C_{13}), 93%	n-undecane (2%), n-dodecane (2%), tridecene (2%), n-pentadecane (0.6%)	
C. gestroi, workers	Israel	5-methyl-Tridecane, 30.3%	n-undecane (11.6%), n-tridecane (21%), n-pentadecane (15.2%), n-hexadecane (8.9%), n-heptadecane (12.6%); trace n-dodecane and n-pentadecene.	
C. aethiops, workers	Corsica	n-Undecane (C_{11}), 72%	n-tridecane (18.1%), n-decane (1.41%), n-dodecane (0.46%), n-pentadecane (6.86%)	Ali *et al.* (1988a)
C. aethiops, queens		n-Undecane (C_{11}), 77.7%	n-tridecane (19.5%), n-decane (1.97%), n-dodecaen (0.25%), trace n-pentadecane	
C. aegyptiacus, minor workers	Egypt	n-Undecane (C_{11}), 78.4%	n-Tridecane (9%), n-decane (3%), dodecyl acetate (0.2%)	Ali *et al.* (1988b)
C. aegyptiacus, major workers		n-Undecane (C_{11}), 52.2%,	mainly hydrocarbons (C_{10} to C_{19}) and acetates (C_{10} to C_{20}); trace compounds of two	

Species and castes	Origin	Main compound (%)	Others	Reference
		Dodecyl acetate (28.2%)	alcohols, two propionates and one terpenoid compound (farnesyl acetate). Different from minors, n-tridecane (3.9%) and n-decane (0.5%), dodecyl acetate (28.2%).	
C. vagus, workers	Corsica	n-Undecane (C_{11}), 70% n-Tridecane (C_{13}), 22%	99% of contents is alkanes (C_9 to C_{15}) and alkenes (C_{11} to C_{19}); small quantities of three methyl-branched alkanes, four acetates and dodecanol.	
C. lateralis, workers	France	n-Tridecane (C_{13}), 73.9%	23 substances were recognized, chiefly alkanes and alkyl acetates, n-tridecane (73.9%), followed by 5-pentadecene (13.25%) and tetradecyl acetate (17.26%); small quantities of a farnesene isomer and geranylgeranyl acetate.	Bagnères *et al*. (1991)
*C. atriceps,*workers	USA	n-Undecane (C_{11}), 76.24%	33 hydrocarbons (C_{10} to C_{21}); trace amount of the doubly unsaturated straight chain tridecadiene, heptadecadiene and nonadecadiene.	Haak *et al*. (1996)
C. floridanus, workers		n-Undecane (C_{11}), 90%	Hydrocarbons (C_{10} to C_{19}); small quantities of monomethyl alkanes and two oxygenated compounds (9-octadecenol and 2,3-dihydro-6t-farnesal)	
C. arminius, workers	South Africa	n-Undecane (C_{11}), 67% n-Tridecane (C_{13}) 19%	Other hydrocarbons (C_{10} to C_{17}), two terpenoid compounds (geranyllinalool and geranylfarnesol), and trace amount of tridecene.	Brand *et al*. (1999)
C. socius, workers	USA	n-Undecane (C_{11}), 93%	The straight chain saturated hydrocarbons (C_9 to C_{19}, and C_{21}) with n-undecane 93%; corresponding mono-unsaturated, straight chain hydrocarbons (C_{11} to C_{13} and C_{16} to C_{20}).	Kohl *et al*. (2001)
C. castaneus, workers	USA	n-Undecane (C_{11}), 95%	mono-unsaturated, straight chain hydrocarbons (C_{12} to C_{13}, and C_{16} to C_{19}), and a small amount of a nonadecadiene.	
C. balzani, workers	Peru	n-Octyl hexanoate, 97%	consist mainly of esters, and Octyl hexanoate makes up about 97% of the total volatile.	Kohl *et al*. (2003)
C. sericeiventris, workers	Argentina	n-Undecane (C_{11}), 84%	mono-unsaturated, straight chain hydrocarbons in the ranges C_{11} to C_{19} (with exception of C_{14} to C_{18}), and also a small amount of 6, 9-heptadecadiene.	
*C. obscuripes,*workers	Japan	n-Undecane(C_{11}), >90%	n-decane and n-pentadecane	Fujiwara-Tsujii *et al*. 2006
